# Plastoquinone-Derivative SkQ1 Improved the Biliary Intraepithelial Neoplasia during Liver Fluke Infection

**DOI:** 10.3390/cimb46020103

**Published:** 2024-02-17

**Authors:** Oxana Zaparina, Anna Kovner, Viktoria Petrova, Nataliya Kolosova, Viatcheslav Mordvinov, Maria Pakharukova

**Affiliations:** 1 Institute of Cytology and Genetics, Siberian Branch of Russian Academy of Sciences (ICG SB RAS), 10 Lavrentiev Ave., Novosibirsk 630090, Russia; zp.oksana.93@gmail.com (O.Z.);; 2 Department of Biology, Cherepovets State University, 5, St. Lunacharsky, Cherepovets 162600, Russia; 3 Department of Natural Sciences, Novosibirsk State University, 2 Pirogova Str., Novosibirsk 630090, Russia

**Keywords:** oxidative stress, liver fluke, chronic inflammation, bile ducts

## Abstract

Carcinogenic food-borne liver fluke infections are a serious epidemiological threat worldwide. The major complications of *Opisthorchis felineus* infection are chronic inflammation and biliary intraepithelial neoplasia. Although evidence has accumulated that increased reactive oxygen species production is observed in liver fluke infection, a direct relationship between the oxidative stress and biliary intraepithelial neoplasia has not been shown. Quinones and SkQ1, a derivative of plastoquinone, have been demonstrated to be cytoprotective in numerous liver injuries due to their potent antioxidant properties. This study is aimed to assess the level of biliary intraepithelial neoplasia in *O. felineus*-infected hamsters after treatment with mitochondria-targeted SkQ1. SkQ1 significantly reduced the biliary intraepithelial neoplasia, which was accompanied by a decrease in lipid and DNA oxidation byproducts, mRNA expression and level of proteins associated with inflammation (TNF-α, CD68) and fibrogenesis (CK7, αSMA), and was also associated with an activation of the Keap1-Nrf2 pathway. Thus, a direct relationship was found between oxidative stress and the severity of biliary intraepithelial neoplasia in *O. felineus*-infected hamsters. The hepatoprotective effect of plastoquinone-derivative SkQ1 was established; therefore, this compound is a promising agent in complex therapy in the treatment of opisthorchiasis.

## 1. Introduction

The liver fluke *Opisthorchis felineus* (Rivolta, 1884) is a trematode of the family Opisthorchiidae that infests the hepatobiliary system of humans and other fish-eating mammals [1]. The main epidemiological area of the infection caused by *O. felineus* is located in Western Siberia, but small foci were also found in some European countries [2], while the foci of this disease tend to expand [1,3]. Opisthorchiasis is accompanied by liver morbidities, including chronic inflammation in the liver, periductal fibrosis, proliferation of the bile ducts, as well as the development of biliary intraepithelial neoplasia (BiliN) [4].

BiliN is considered a precursor to cholangiocarcinoma, a premalignant, noninvasive neoplastic lesion of the gallbladder or bile duct. BiliN grades I, II and III have been reported in hamsters infected with *O. felineus* [4]. Related species of the family Opisthorchiidae, *O. viverrini* (Poirier, 1886) and *C. sinensis* (Looss, 1907), are recognized as biological carcinogens and the main factors of cholangiocarcinoma in endemic areas [5,6]. The mechanisms of BiliN development in liver fluke infections remain elusive; however, the time-dependent accumulation of oxidative damage, such as lipid peroxidation byproducts, has been revealed [7,8,9]. Moreover, increased levels of oxidative DNA byproducts, such as 8-oxo 2-deoxyguanosine (8-OHdG), are observed during liver fluke infection [8,10]. Thus, elevated concentrations of 8-oxodG in urine are positively associated with advanced periductal fibrosis and cholangiocarcinoma in liver-fluke-infected patients [10]. Therefore, oxidative stress plays an important role in the pathogenesis of liver fluke infections. The relationship between BiliN and the level of oxidative stress has not been proven; moreover, the mechanisms of development of precancerous changes during biological carcinogenesis, in particular during carcinogenesis caused by liver flukes, have been poorly studied.

Nevertheless, some indirect evidence of the involvement of oxidative stress in structural changes in the liver suggests that inhibition of free radical excess may have a positive effect on the level of BiliN in opisthorchiasis. However, a direct relationship between the level of epithelial neoplasia and redox status has not been shown. In order to test this hypothesis, it was decided to carry out an inhibitory assay using an antioxidant. Positive effects of antioxidants on reducing inflammation and altered redox status in the liver during liver fluke infections have been shown. This was primarily related to inflammation and the periductal fibrosis area [11,12]. 

Quinones and their chemical derivatives have been proven to be effective for the pharmacotherapy of many liver injuries as bioactive antioxidants [13,14,15]. One such compound is the plastoquinone derivative SkQ1. SkQ1(-10-(6′-Plastoquinonyl) decyltriphenylphosphonium) is a mitochondria-targeted antioxidant, a chemical compound consisting of the antioxidant plastoquinone, a lipophilic cation and a decamethylene linker. Previously, we have demonstrated that SkQ1 is able to ameliorate host oxidative stress during early infection in hamsters [8]. The choice of SkQ1 for this study was due to the following benefits: it has a known direct antioxidant mechanism [16], it exhibits activity in low doses [17] and it has mild side effects with long-term use [18].

In this research, we assessed the effect of the mitochondria-targeted antioxidant SkQ1 on the level of BiliN, the area of periductal fibrosis, the level of lipid and DNA oxidative byproducts in a model of experimental opisthorchiasis on golden hamsters *Mesocricetus auratus* at 1 and 3 months of a diet with the antioxidant. Additionally, we assessed the expression of mRNA genes and the production of inflammatory and fibrogenesis-related proteins. Moreover, we also evaluated the anthelmintic activity of this mitochondria-targeted antioxidant in vivo.

## 2. Materials and Methods

### 2.1. Ethics Statement

All the procedures were in compliance with European Union Directive 2010/63/EU for animal experiments. The animals were kept and treated according to the protocols approved by the Committee on the Ethics of Animal Experiments at the Institute of Cytology and Genetics, the Siberian Branch of the Russian Academy of Sciences, Russia (ICG SB RAS; Permit Number: 42 of 25 May 2018).

### 2.2. Compounds and Doses

SkQ1 (10-(6′-Plastoquinonyl) decyltriphenylphosphonium) (Institute of Mitoengineering of Moscow State University, Russia) was dissolved in ethanol to prepare a 162 µmol/mL solution. Before oral administration, it was further diluted with water (50 µL to 1 mL H_2_O) to final concentration 8 µmol/mL.

PZQ was prepared as a suspension in 7% Tween 80 (*v*/*v*) and 3% ethanol (*v*/*v*) before oral administration (10 mL/kg). For resveratrol (RSV) administration to uninfected animals, RSV (Sigma, St. Louis, MO, USA) was dissolved in ethanol to prepare an 88 mg/mL solution. Before oral administration, it was diluted with PBS (pH 6.11) to a final concentration of 1 mg/mL.

### 2.3. Animals and Experimental Design

All experiments were conducted on male Syrian hamsters M. auratus obtained from the Animal Facility of the ICG SB RAS, in accordance with EU Directive 2010/63/EU for animal experiments. Hamsters were kept under standard conditions and received food for rodents (PK-120-1; Laboratorsnab, LLC, Moscow, Russia) and water ad libitum. The hamsters were euthanized by carbon dioxide inhalation and every effort was made to minimize suffering.

Metacercariae of *O. felineus* were isolated from the subcutaneous muscle tissue of infected fish (ides) caught in the Ob River in the vicinity of Novosibirsk city (Western Siberia) and extracted accordingly [4,19].

**Figure 1:**
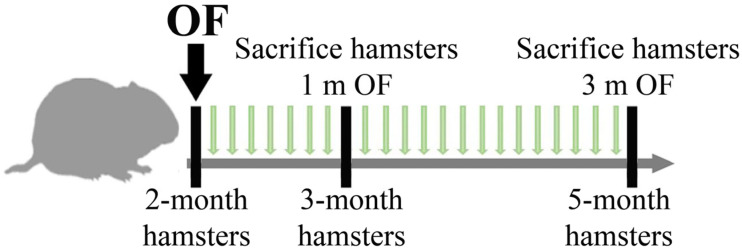
A scheme showing the treatment schedule of hamsters. Animals received a daily food supplement of antioxidant 10-(6′-Plastoquinonyl)decyltriphenylphosphonium [SkQ1]) within 1 and 3 months.

Thirty-five 2-month-old hamsters were randomly divided into 4 groups (1 and 3 months of infection): (I) control group, (II) uninfected group receiving dietary SkQ1 (1 µmol/kg), (III) animals infected with *O. felineus* and (IV) animals infected with *O. felineus* and fed SkQ1 (1 µmol/kg). Hamsters of groups III and IV were infected orally using a gastric tube (Figure 1).

Animals from Groups I and III (without SkQ1) received a piece of dried bread (5 × 5 square mm) daily, in addition to the main diet, from the first day of infection; other groups received dried bread with the addition of SkQ1. The total duration of the experiment was 3 months. Animals were sacrificed at 1 and 3 months post-infection (p.i.). As a positive control to assess the activation of NRF2/NQO1, an additional group (5 animals) was added: uninfected animals that received resveratrol daily in the diet at a concentration of 1 mg/kg for 1 month.

### 2.4. In Vivo Chemotherapy

Thirteen 2-month old male hamsters were infected with 75 metacercariae. The animals from the group (*O. felineus* + SkQ1) had the same diet with SkQ1 (1 micromol/kg) as that from Group II and Group IV. Hamsters from the *O. felineus* + PZQ group were treated with a single oral 400 mg/(kg body weight) praziquantel dose via oral administration at 5 weeks p.i., as described earlier [4,19].

Worms remaining in the hepatobiliary system on day 7 post-treatment were counted. Drug activity was expressed as a worm burden reduction (WBR) as described elsewhere [19,20]. Briefly, WBRs were calculated as follows: ((a − b)/a) × 100, where a is the average worm count in the control group upon dissection, and b denotes the average worm count in the treated group upon dissection. The significance of WBR was evaluated by the Mann–Whitney U test in the STATISTICA 6.0 software (StatSoft, Tulsa, OK, USA) [19].

### 2.5. Material Collection, Histopathology and Immunohistochemistry

Blood was collected by cardiac puncture. To obtain serum, the blood was centrifuged at 3000× *g* and a temperature of +4 °C for 20 min. Urine was collected by bladder puncture. The resulting serum and urine were divided into aliquots and stored at −80 °C. To carry out histological analysis, the liver was fixed in buffered 10% formalin (Biovitrum, Saint Petersburg, Russia), and standard histological analysis was performed with hematoxylin-eosin and Masson’s trichrome staining, as described previously [4,19].

The assessment of pathological changes by semiquantitative scoring system of type ratio (morphometry) [20] was carried out as described previously [8]. In particular, all visual fields were analyzed (20–30 images) from two liver lobes of each animal. Each field of view was divided into 100 squares by a morphometric grid. Data were calculated using the ImageJ program. The following were assessed: inflammatory infiltration, periductal fibrosis, hyperplasia, BiliN, proliferation of the bile ducts and parenchymal dystrophy. Immunohistochemical analysis was performed (SpringBioScience, Pleasanton, CA, USA; cat. # HRP-125) using specific primary antibodies: NRF2 (1:100; cat. #ab62352, Abcam, Cambridge, UK) and NQO1 (1:100; cat. # ab213239, Abcam, Cambridge, UK) according to the manufacturer’s protocol.

For immunohistochemical analysis of cryosections, the liver was fixed in buffered 4% paraformaldehyde at +4 °C for 12 h, then fixed in 30% sucrose for 24 h at 4 °C and stored at −80 °C. Cryosections (7–10 μm) were prepared on a Microm HM-505 N. Immunohistochemical analysis was carried out in accordance with the standard protocol described previously [4]. Antibodies and dilutions used in this study include anti-4 hydroxynonenal antibody (1:150; cat. # ab48506, Abcam, Cambridge, UK) and anti-malondialdehyde antibody (1:300; cat. # ab6463, Abcam, Cambridge, UK), followed by Second antibodies Invitrogen Goat anti-Mouse IgG (H + L) Cross-Adsorbed Secondary Antibody, Cyanine3 (1:500; cat. #M30010) and AffiniPure Goat Anti-Rabbit IgG (H + L) (1:1000 cat # 111-095-003). DAPI was applied for nuclear staining.

### 2.6. Serum Biochemistry

Serum biochemical values were measured (activity of ALT (alanine aminotransferase), AST (aspartate aminotransferase), the content of total protein, bilirubin, cholesterol, creatinine, urea and triglycerides) using commercially available kits (Vector-Best, Moscow, Russia) in accordance with the manufacturer’s recommendations. The data obtained were compared with normal physiological parameters of laboratory animals [21]. Urinary 8-hydroxy-20-deoxyguanosine (8OH-dG) concentration was measured using the 8OH-dG ELISA kit (cat. # ab201734, Abcam, Cambridge, UK).

### 2.7. Total RNA Extraction, cDNA Synthesis and Real-Time PCR

Total RNA was isolated from the liver using the Aurum total RNA extraction kit (Bio-Rad, Hercules, CA, USA). Concentrations of RNA were determined on a NanoDrop spectrophotometer (ND1000, NanoDrop Technologies, Wilmington, DE USA). First-strand cDNA synthesis was performed with the RevertAid Kit (Thermo Fisher Scientific, Waltham, Massachusetts, USA). Expression levels of the genes were measured by real-time PCR using the EVA Green Reagent Mix (Synthol, Moscow, Russia) on a CFX96 real-time PCR system (Bio-Rad, Hercules, CA, USA). Sequences for all primers and probes are given in Supplementary Table S1 (Synthol, Moscow, Russia). As an endogenous internal control for normalization, we chose Gapdh. Triplicate real-time PCRs were performed on each sample. Data analysis was performed using CFX96 Manager 3.1 (Bio-Rad, Hercules, CA, USA).

### 2.8. Western Blotting

To prepare liver lysates, a small piece of liver (1 × 2 mm) was homogenized using a plastic homogenizer in 300 μL of RIPA lysis buffer (150 mM NaCl, 50 mM Tris HCl pH 8.0, 0.1% SDS, 0.1% Triton X-100) + 1 mM PMSF (Phenylmethylsulfonyl fluoride) and sonicated (10 s, 2 times, 30% amplitude) (Vibra-Cell VCX130) on ice. Then, the protease inhibitor cocktail from Amresco (AEBSF, Aprotinin, E-64, Bestatin and Leupeptin) was added, and the tube was placed on a rotator for 1.5–2 h at +4 °C. Next, the lysates were centrifuged for 20 min at 12.5 rpm at +4 °C. The supernatant was aliquoted and frozen at −80 °C.

Human HepG2 hepatoma cells (kindly provided by T.A. Schneider, Institute of Cytology and Genetics SB RAS, Novosibirsk, Russia) were grown in a medium containing DMEM/F12, 10% FBS, 100 IU/mL of penicillin and 100 μg/mL of streptomycin. A total of 4 × 10^3^ cells were seeded in a 6-well plate and expanded to 80% monolayer. SkQ1 was added to a well at a concentration of 1 µM, dissolved in ethanol and cultured for 4 h and 24 h (in triplicates). Control cells were incubated with 0.2% ethanol. As a positive control, cells were incubated for 4 h with 2.5 mM resveratrol. To obtain a cell lysate, 1 mL of ice-cold PBS was added to the cell pellet and centrifuged for 5 min at 400× *g* at +4 °C, then 100 µL RIPA buffer was added and resuspended. Next, the cells were homogenized using a syringe and incubated on ice for 30 min. Then, the tube was centrifuged for 10 min at 12,000 rpm at +40 °C. The protease inhibitor cocktail from Amresco (AEBSF, Aprotinin, E-64, Bestatin and Leupeptin) was added to the supernatant. The supernatant was divided into aliquots and frozen at −80 °C. Protein concentration was determined using the BCA Protein Assay kit (Thermo Fisher Scientific, Waltham, MA, USA) according to the manufacturer’s protocol.

Immunoblotting was performed as described elsewhere [4]. Antibodies and dilutions used in this study include β-actin (1:2000; cat. # ab8226, Abcam), smooth muscle actin a (1:2000; cat. # ab7817, Abcam), cytokeratin 7 (1:2000; cat. # ab9021, Abcam), TNF-a (1: 2000; cat. # 3114560, Sony), CD68 (1: 2000; cat. # ab201340, Abcam), TGF-β (1: 2000; cat. #), Nrf2 (1:4000; cat. # ab62352, Abcam) and NQO1 (1:4000; cat. # ab213239, Abcam) antibodies and secondary antibodies conjugated to horseradish peroxidase (Gt Anti-Rb IgG (HRP) cat. No. ab 205718 Abcam; Rb Anti-Ms IgG (HRP) cat. No. 1114. SantaCruz, Santa Cruz, CA, USA), at a dilution of 1:20 000. The signal was detected using the ECL chemiluminescent reaction reagent (Amersham Biosciences, Little Chalfont, UK). Quantitative densitometry was performed on digitized images of immunoblots in the Quantity One version 4.6.9 (Bio-Rad, Hercules, CA, USA).

### 2.9. Statistics

Statistical analyses were performed with the Statistica 6.0 software (Statsoft, Tulsa, OK, USA). RT-PCR data were subjected ANOVA to the F-test with the Tukey post hoc analysis. Significance of the differences between the groups of hamsters was evaluated by the Mann–Whitney test (quantitative histopathology and serum biochemical parameters). Normality of distribution was determined using the Shapiro–Wilcoxon W test.

## 3. Results

### 3.1. Liver Histopathology

**Figure 2:**
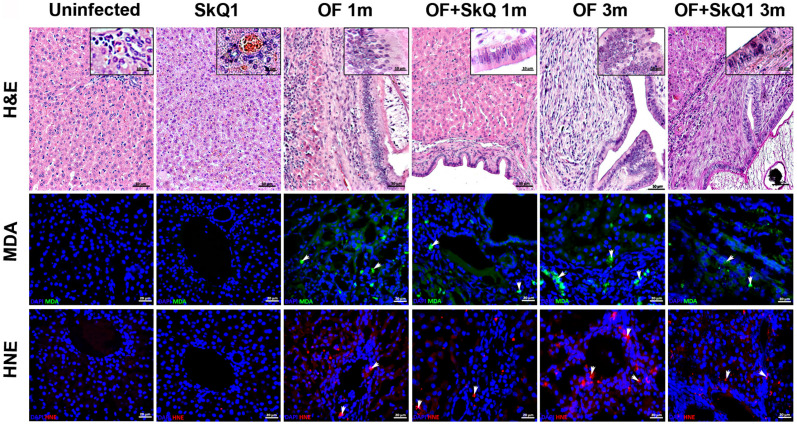
Liver sections stained with hematoxylin and eosin (H&E, upper panel); accumulation of peroxidation markers MDA (Malondialdehyde, middle panel) and HNE (4-hydroxynonenal, lower panel). MDA (green) specific signal and HNE (red) specific signal are indicated with arrows. DAPI (blue); BD—bile duct; BV—blood vessel; PF—periductal fibrosis; II—inflammatory cell infiltration.

In the infected animals, the dilated bile ducts were noted, around which a huge area of periductal fibrosis was observed (Figure 2; Supplementary Figure S1). The foci of inflammation were seen in the portal area of the liver, with the infiltration with mononuclear cells. Moreover, the pronounced proliferation of bile ducts, structural changes in parenchyma, including modification of the shape and structure of nuclei, the formation of large vacuoles in the cell and the emergence of uncharacteristic inclusions were noted (dystrophy of parenchyma). Cholangiocyte hyperplasia was noted at 1 and 3 months p.i., and biliary intraepithelial neoplasia was seen at 3 months p.i.

**Figure 3:**
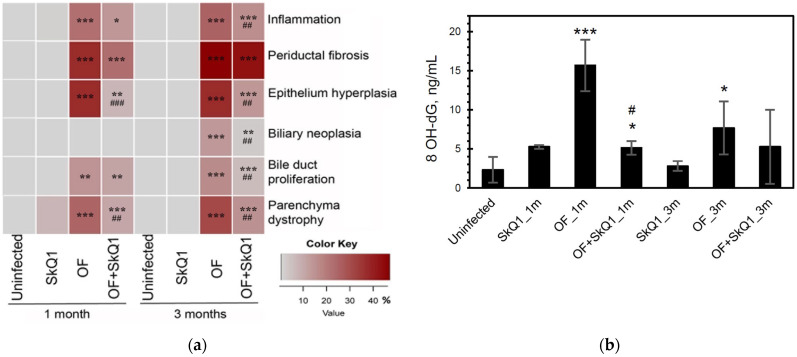
Quantitative histopathology analysis and urinary 8-hydroxy-2′-deoxyguanosine content. (**a**) Histopathology data are expressed as a percentage of the maximum possible score and presented as a heat map. OF—group infected with *O. felineus*; OF + SkQ1—infected group treated with SkQ1. (**b**) 8-hydroxy-2′-deoxyguanosine (8-OHdG) in the urine of hamsters. *—compared to the uninfected group, #—compared to the OF-infected group; ANOVA + post hoc, mean ± SD. *, # *p* < 0.05; ##, ** *p* < 0.01; ###, *** *p* < 0.001.

SkQ1 significantly alleviated the level of pathological manifestations already at 1 month p.i. (Figure 2 and Figure 3a, Supplementary Table S2). However, the most pronounced effect of the antioxidant was observed at 3 months p.i. In particular, the degree of inflammation in animals (Group IV) was 1.6 times less than that of Group III (OF-infected hamsters) (*p* = 0.01), whereas the cholangiocyte hyperplasia was decreased by 1.4 times (*p* = 0.009). An important result was a decrease in the level of neoplasia of the bile duct epithelium by 1.6 times (*p* = 0.008), the degree of proliferation of the bile ducts by 3 times (*p* = 0.04), while the level of periductal fibrosis was not significantly alleviated by the antioxidant. SkQ1 improved the structure of liver parenchyma and reduced the liver parenchyma dystrophy by two times at 1 and 3 months p.i. (*p* = 0.01). It was shown that SkQ1 did not affect the liver structure of uninfected animals (Group II, Figure 2). The results of the semiquantitative histological analysis are presented as a heat map in Figure 3a and Supplementary Table S2.

### 3.2. Oxidative Lesions

Accumulation of lipid peroxidation byproducts MDA (malondialdehyde) and HNE (4-hydroxy-2-nonenal) was detected at 1 month p.i., mainly in the area of the bile duct epithelium. Later, MDA and HNE signals increased and were noted in the area of periductal fibrosis and liver parenchyma at 3 months p.i., which is consistent with previously obtained data [5]. In the liver of animals of Group IV (OF + SkQ1), there was a decrease in the specific signal of MDA and HNE at 1 and 3 months p.i. (Figure 2). In the control groups (Group I and II), no accumulation of MDA and HNE was observed.

The level of urinary oxidative stress marker 8-OhdG increased in the infected animals (Group III) starting from 1 month p.i. (6.8-fold; *p* = 0.001) and remained elevated at 3 months p.i. (3.3-fold; *p* = 0.04). The antioxidant SkQ1 significantly reduced 8-OhdG levels at 1 month p.i. (3-fold; *p* = 0.03), but after 3 months, the effect of the antioxidant was insignificant (Figure 3b).

### 3.3. Fibrosis- and Inflammation-Related Gene Expression

**Figure 4:**
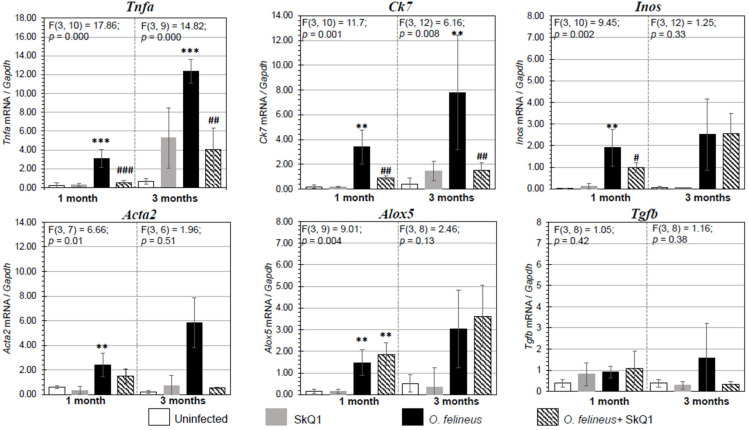
Expression of mRNA of inflammation- and fibrogenesis-related genes proteins in the liver of golden hamsters *Mesocricetus auratus* at 1 and 3 months p.i. The data are presented as the fold change normalized to the *Gapdh* mRNA level. *Alox5*: lipoxygenase 5; *Tnfa*: tumor necrosis factor α; *Acta2*: smooth muscle actin α; *Ck7*: cytokeratin 7; *Tgfb*: transforming growth factor β; *Inos*: nitric oxide synthase. # *p* < 0.05; ##, ** *p* < 0.01; ###, *** *p* < 0.001; ANOVA + post hoc, mean ± SD. *—compared to the uninfected group, #—compared to the *O. felineus*-infected group.

There was a significant increase in the expression of the genes *Tnfa* (tumor necrosis factor α), *Alox5* (lipoxygenase 5), *Inos* (inducible nitric oxide synthase), *Acta2* (smooth muscle actin α) and *Ck7* (cytokeratin 7), associated with the regulation of inflammation and fibrogenesis in hamsters infected with *O. felineus* (Group III) (Figure 4). The mRNA expression levels of genes involved in inflammation and fibrogenesis were increased in *O. felineus*-infected groups at 1 and 3 months p.i. The increase in *Tnfa* mRNA level in the infected group (Group III) was 6 and 11 times (*p* = 0.001) at 1 and 3 p.i., respectively. Expression levels of *Alox5* and *Inos* were also significantly increased at 1 month p.i. (*p* = 0.004 and *p* = 0.002, respectively).

**Figure 5:**
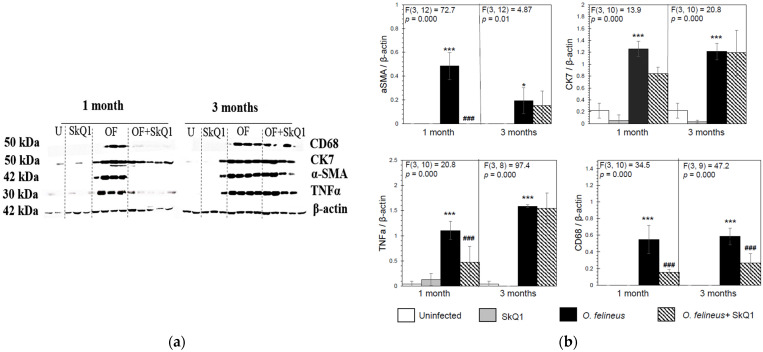
Content of inflammation- and fibrogenesis-related proteins in the liver of golden hamsters *Mesocricetus auratus* at 1 and 3 months p.i. (**a**) Representative immunoblots are shown; (**b**) densitometry of immunoblots. Quantification of the intensity of the protein is presented normalized to β-actin. * *p* < 0.05; ###, *** *p* < 0.001; ANOVA + post hoc, mean ± SD. *—compared to the uninfected group, ###—compared to the *O. felineus*-infected group.

Antioxidant SkQ1 in Group IV significantly alleviated the level of expression of *Tnfa* (threefold; *p* = 0.001 at 1 month p.i. and twofold; *p* = 0.01 at 3 months p.i.) and *Inos* (twofold; *p* = 0.02) at 1 month p.i. and had no effect on *Alox5* mRNA expression. The expression of *Acta2* mRNA under the influence of SkQ1 was reduced (twofold after 1 month p.i., *p* = 0.02), while SkQ1 revealed its effect to a greater extent at 3 months p.i., reducing the *Acta2* mRNA level by 24 times (*p* = 0.037). *Tgfb* (transforming growth factor β) mRNA expression was increased in OF-infected animals (Group III) at 1 and 3 months p.i. (*p* = 0.04) and was not affected by the antioxidant (Group IV). The mRNA level of *Tnfa*, *Alox5*, *Inos*, *Acta2*, *Ck7* and *Tgfb* in the control groups (Groups I and II) was negligible (Figure 4). Thus, SkQ1 suppressed the expression of inflammation- and fibrogenesis-related genes. These results correlate with the data of Western blot analysis (Figure 5a,b) and histological studies and confirm that a diet with SkQ1 leads to alleviation of the severity of opisthorchiasis-related morbidities.

### 3.4. NRF2 and NQO1

**Figure 6:**
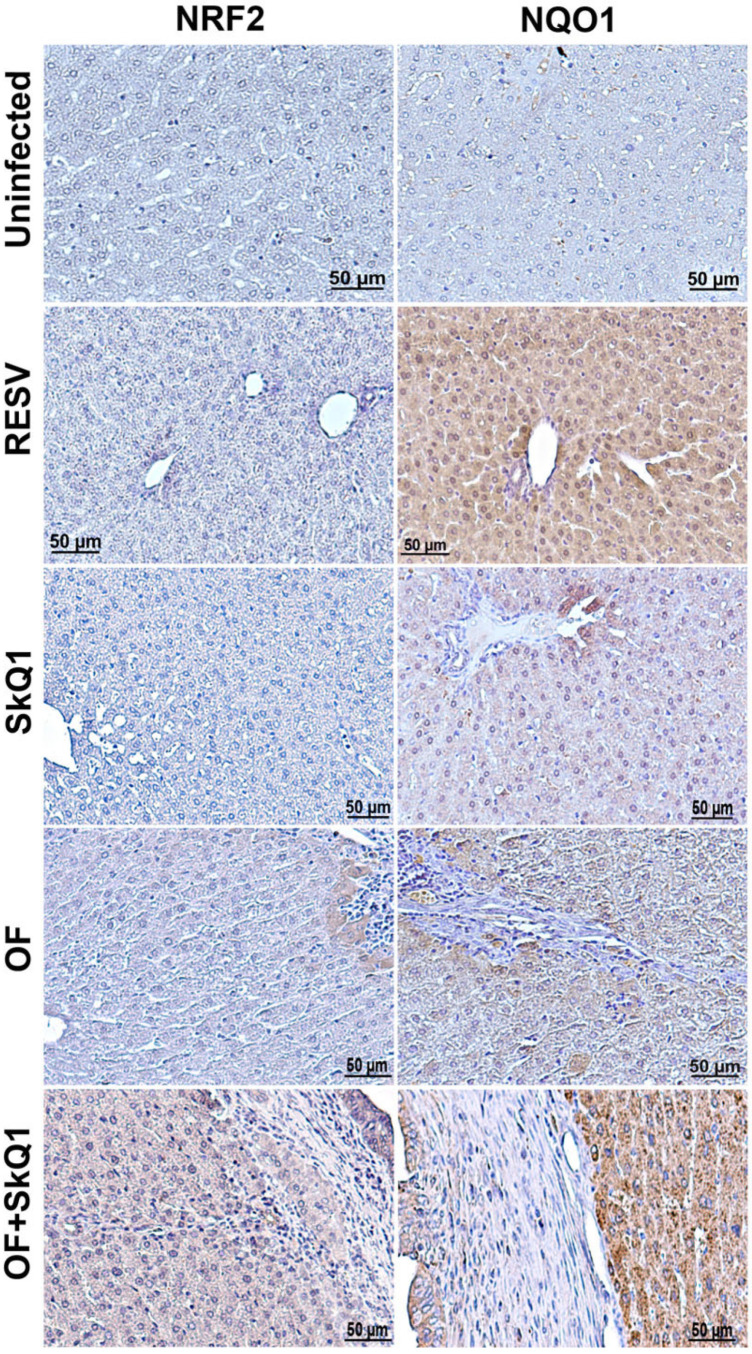
Immunohistochemistry of liver tissue from hamsters at 1 month of *O. felineus* infection. SkQ1—uninfected, treated with SkQ1 antioxidant; RESV—uninfected, treated with resveratrol; OF—infected with *O. felineus*; OF + SkQ1—infected with *O. felineus* and treated with SkQ1.

The protective effect of SkQ1 during oxidative stress can probably be realized not only through direct antioxidant activity but also as a result of activation of the Keap1/Nrf2/ARE signaling pathway [22]. In order to investigate the possible activation of the NRF2 transcription factor by SkQ1, we examined the level of NRF2 and NQO1 (NAD(P)H quinone dehydrogenase-1), an NRF2 target gene, in the liver of uninfected and infected animals (Figure 6). Resveratrol was used as a positive control because it was found that resveratrol modulates NRF2 activation [23].

Indeed, the NQO1 level was increased after the resveratrol treatment in the liver of uninfected animals (Figure 6). We also found that SkQ1 in uninfected animals increases the level of NQO1 in hepatocytes to a lesser extent (Group II). In groups infected with *O. felineus*, a slight increase in the content of both NQO1 and NRF2 was noted (Groups III and IV). In this case, a specific signal was noted in the cytoplasm of hepatocytes located in close proximity to the foci of inflammation and proliferation of the bile ducts. In the group of *O. felineus*-infected and SkQ1-treated animals (Group IV), the signal of NQO1 and NRF2 was significantly increased and noted not only in hepatocytes but also in the bile duct epithelium (Figure 6).

**Figure 7:**
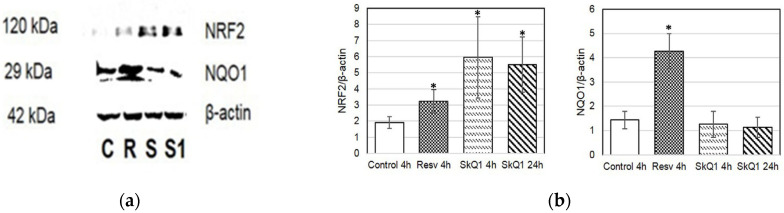
Western blot analysis of HepG2 cells treated with resveratrol (R) and SkQ1 (S) for 4 h or 24 h (S1, SkQ1-treated). (**a**) Representative immunoblots are shown; (**b**) densitometry of immunoblots. Quantification of the intensity of the protein is presented normalized to β-actin. C—control. * *p* < 0.05 as compared to the control group; ANOVA + post hoc, mean ± SD.

Additionally, to investigate the activation of proteins involved in the ARE-dependent pathway, we examined the protein levels of NQO1 and NRF2 in HepG2 cell culture (Figure 7). The highest levels of NRF2 were observed in cells treated with SkQ1 at 4 and 24 h (Figure 7a,b).

### 3.5. Evaluation of Anthelmintic Properties of SkQ1 In Vivo

The significant decrease in the level of biliary intraepithelial neoplasia that we observed, as well as a decrease in gene expression and production of pro-inflammatory proteins, can be explained, among other things, by a decrease in the level of parasitic load in hamsters. In addition, preliminary data showed that SkQ1 affects the motility of juvenile and adult *O. felineus* worms in vitro [24]. Therefore, we assessed the anthelmintic activity of SkQ1 in vivo. The anthelmintic drug praziquantel was taken as a positive control.

Unlike praziquantel, which resulted in a 76.5% reduction in total worm burden, SkQ1 treatment did not result in a worm burden reduction. Thus, SkQ1 does not have anthelmintic activity in vivo (Table 1). Consequently, the data on a decrease in the severity of pathological processes in the animals from Group IV cannot be explained by a decrease in the parasitic load caused by a diet with SkQ1.

### 3.6. Serum Biochemistry

An increase of the following biochemical parameters was found in the infected animals (Group III) compared with Group I: the activity of ALT (6 times and 10 times higher; *p* = 0.02 at 1 and 3 months p.i., respectively), AST (1.8 times higher; *p* = 0.03 at 3 months p.i.), total bilirubin level (3.6 times elevated; *p* = 0.02 and 2.8 times; *p* = 0.01 at 1 and 3 months p.i., respectively) and cholesterol level (1.8 times; *p* = 0.02 at 1 and 3 months p.i.). The data are in line with our previous results, which confirm the data about liver injury in opisthorchiasis. After SkQ1 administration (Group II), we found an increase in total bilirubin (1.7 times; *p* = 0.03) at 1 month p.i., but no side effects have been shown for SkQ1 at this concentration [25]. The level of serum urea was also significantly increased (1.9 times; *p* = 0.02) compared to that in Group I but fell within the reference values. Interestingly, these values in Group II did not change from those in Group I at 3 months p.i.

After the SkQ1 diet, there was a trend toward decreasing ALT, AST, total bilirubin and cholesterol levels. Nevertheless, the level of triglycerides in Group IV at 3 months p.i. was three times higher (*p* = 0.01) than that in Group III. The data are presented in Supplementary Table S3.

## 4. Discussion

In this study, we have demonstrated for the first time that the level of oxidative stress markers in the liver during opisthorchiasis directly correlates with the level of BiliN. This was demonstrated using the mitochondria-targeted plastoquinone SkQ1, which reduces the accumulation of key markers of oxidative stress (MDA, HNE and 8 OH-dG) and, in addition, reduces the area of BiliN and the level of expression of inflammation- and fibrogenesis-related genes.

Opisthorchiasis, caused by infection with the trematode *O. felineus*, is accompanied by various structural and functional hepatobiliary morbidities. Such disorders include changes in the activity of liver transaminases, increased bilirubin levels, the formation of foci of chronic inflammation, the formation of periductal fibrosis, cholangiofibrosis, hyperplasia, metaplasia and BiliN of different grades [2,4]. Neoplasia is a pathological process that is accompanied by highly elevated cell proliferation processes, atypical transformation, loss of connection with the basement membrane and is considered precancerous. The accumulation of oxidative byproducts such as malondialdehyde (MDA) and 4-hydroxy-2 nonenal (HNE) has been previously revealed in several studies [7,9]. During chronic inflammation, immune cells are deployed to sites of injury, thereby increasing the release and accumulation of free radicals. Free radicals, in turn, are capable of triggering lipid peroxidation with the subsequent formation of oxidative byproducts, which have cytotoxic properties contributing to the development of various pathological injuries [26,27]. In addition, *O. felineus* infection is associated with increased urinary 8-OHdG level, which is one of the predominant forms of oxidative DNA byproducts caused by free radicals [5]. This indicates the presence of oxidative DNA damage induced by free radicals [26]. The totality of the data obtained on the effect of SkQ1 on the level of oxidative stress markers MDA, HNE and 8-OHdG in infected animals is consistent with the results of histological examination and analysis of gene expression presented above and confirm the role of the antioxidant in mitigating the manifestations of liver fluke infection.

Pathological changes of opisthorchiasis are believed to be caused by the mechanical damage produced by helminths, the effect of their secretory–excretory products [3,28,29] and also helminth-associated changes in the host microbiota [30,31,32]. However, the exact mechanisms of BiliN development are still not understood. Thus, the role of oxidative stress is only one of the possible mechanisms. Recently, more and more evidence has emerged that helminths are capable of modifying the host microbiome, which, in turn, can affect the pathogenesis [32]. Thus, changes in the host microbiome during opisthorchiasis caused by *O. viverrini* are associated with the development of cholangiocarcinoma, including due to the potentially carcinogenic effect of the inflammatory response resulting from bacterial translocation [28,30]. Indeed, helminth infections significantly change the host microbiome [30,31,32,33], leading to the emergence and spread of pathogenic bacteria, which likely influences the development of neoplasia. Whether SkQ1 is able to mitigate the consequences of an altered host microbiome during liver fluke infection remains unknown.

SkQ1 is a mitochondrial-targeted antioxidant, which, due to its physicochemical properties, is able to penetrate into mitochondria and neutralize reactive oxygen species directly at the site of their generation. SkQ1 is a lipophilic cation linked via a C-aliphatic (decamethylene) linker to the antioxidant plastoquinone. SkQ1 is capable of being restored by the mitochondrial respiratory chain. Either NAD-bound substrates or succinates are used as electron donors. After neutralization of ROS, the plastoquinone residue passes into an oxidized form. It is then quickly restored by complex III of the respiratory chain. Thus, due to the functioning of the respiratory chain, SkQ1 exists mainly in a reduced state, capable of exhibiting antioxidant activity. Inactivation of reactive oxygen species under the influence of SkQ1 can occur in several ways: due to the oxidation of the plastoquinone and due to a decrease in the mitochondrial membrane potential, the so-called uncoupling of respiration and ATP synthesis [25]. Thus, SkQ1 is able to inhibit ROS, maintain the redox balance in mitochondria and, due to this, reduce the degree of inflammation [34,35]. In addition, SkQ1 has been shown to have beneficial effects in various pathologies [14,36]. Thus, SkQ1 is able to prevent and/or suppress development of all manifestations of accelerated senescence in OXYS rats. Its effects are due to the impact on the several signaling pathways and are associated with restoration of the structural and functional parameters of mitochondria [37].

We hypothesize that the positive effect of SkQ1 may be due to at least two mechanisms: its direct antioxidant effect and through activation of the NRF2-signaling pathway. Nuclear factor-erythroid 2-related factor 2 (NRF2) is considered one of the most significant factors involved in the regulation of the cytoprotective effect. NRF2 is a member of the Cap‘n’Collar family of transcription factors, which are involved in the regulation of genes encoding antioxidant and detoxification enzymes. Thus, the NRF2 pathway serves as the primary mechanism by which the cell can respond to excess accumulation of reactive oxygen species or reactive nitrogen species (ROS/RNS) [38]. Multiple studies have established a connection between NRF2 and various liver pathologies, including alcoholic steatohepatitis [39], non-alcoholic fatty liver disease [40], cirrhosis [41] and the development of hepatocellular carcinoma [42]. In our study, we demonstrated an activation in the NRF2 content. Activation of NRF2 can block inflammation by inhibiting the transcription of pro-inflammatory genes, particularly *Tnfa* and *Inos* (Figure 4), or by inhibiting inflammatory nuclear factor-kappa B signaling activity. Our data are generally consistent with previously published indirect data on the possible activation of the Keap1-Nrf2 signaling pathway by SkQ1. In particular, it was reported that the expression of genes of the antioxidant system (Sod1-2, Cat and Gpx4) increased after pre-treatment with SkQ1 under hypoxic conditions [22]. 

This study also has some limitations. Among these is the lack of assessment of the long-term effect of SkQ1 on BiliN development, limited to only 3 months of infection. Understanding the durability of the therapeutic effects and any potential long-term consequences of the treatment is crucial, especially for a condition that might have chronic implications like BiliN. Nevertheless, our results provide new understanding of the mechanisms of BiliN development in liver fluke infection, and the listed limitations represent an opportunity for further research.

## 5. Conclusions

Oxidative stress has been linked to the development and progression of various disorders. In our study, we showed that the inhibitory assay with the antioxidant SkQ1 resulted in a decrease in BiliN and an improvement in the structural state of the liver of the infected animals. The exact pathways of BiliN development, as well as the molecular mechanisms of biological carcinogenesis, i.e., carcinogenesis induced by liver flukes remain unknown and require further investigation.

This research is focused on the mechanisms of biological carcinogenesis, in particular, on the precancerous changes in the bile duct epithelium and markers of neoplastic processes during infection with liver flukes. Using inhibitory analysis, we were able to show that behind the formation of biliary neoplasia is an increase in oxidative lipids and DNA damage of host cells. Damage occurs as a result of excess of free radicals generated in the liver. The results obtained may contribute to a more complete understanding of the mechanisms that cause pathological changes in opisthorchiasis and will also allow us to consider the possibility of using antioxidants in the combinatorial therapy of helminthiases. At the same time, specific cellular pathways of the emergence and development of precancerous pathological changes during liver fluke infection require further detailed investigation, possibly using cellular models.

## Figures and Tables

**Table 1:** Worm burden reduction values obtained after treatment of hamsters harboring *O. felineus* with SkQ1 or praziquantel in vivo.

	Untreated	SkQ1	Praziquantel
Number of animals	5	3	5
Mean no. of worms ± SD	56 ± 9	55 ± 12	8 ± 6 **
WBR (%)			

Data are presented as mean ± SD (Mann–Whitney U-test); ** *p* < 0.01; WBR (%)—worm burden reduction. WBRs were calculated as follows: ((a − b)/a) × 100, where a is the average worm count in the control group upon dissection, and b denotes the average worm count in the treated group upon dissection.

## Data Availability

All data generated or analyzed during this study are included in this published article.

## Supplementary Materials

The following supporting information can be downloaded at: https://www.mdpi.com/article/10.3390/cimb46020103/s1, Table S1: Primers and probes; Table S2: The results of the semiquantitative histological analysis; Table S3: Serum biochemistry; Figure S1: Masson’s trichrome staining of liver sections to reveal connective tissue (blue).
