# Supplementary material for: Plastoquinone-Derivative SkQ1 Improved the Biliary Intraepithelial Neoplasia during Liver Fluke Infection
Source: Curr Issues Mol Biol. 2024 Feb 17;46(2):1593–606. doi: 10.3390/cimb46020103 (PMC10887543; doi:10.3390/cimb46020103)
Supplement: Supplementary file 1 [file cimb-46-00103-s001.zip › Supplementary Figure S1.pdf]

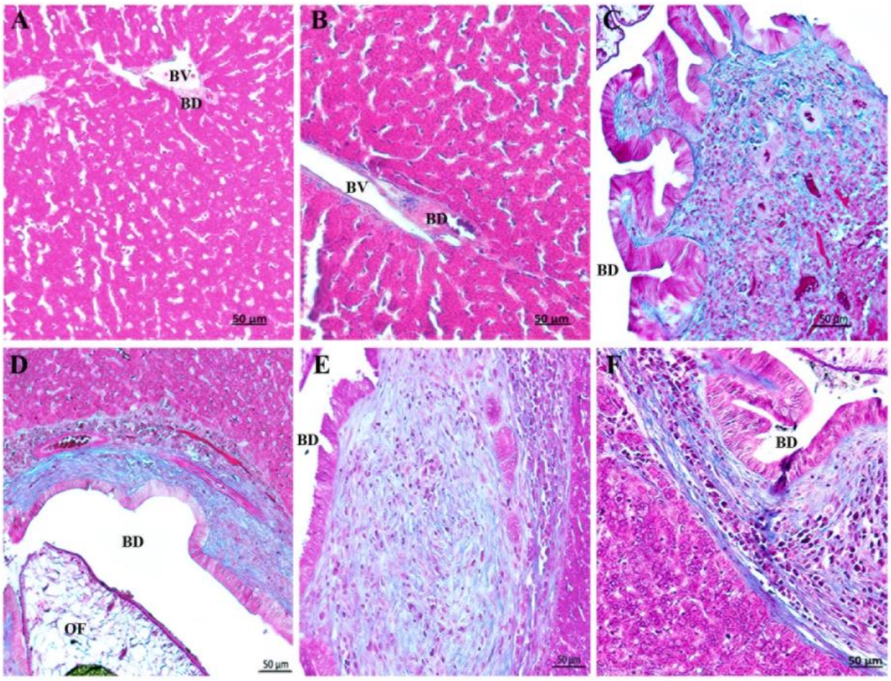

Supplementary Figure S1. Masson's Trichrome staining of liver sections to reveal connective tissue (blue). Representative images are presented. **A** – uninfected; **B** - uninfected, SkQ1 treated; **C** - *O. felineus*-infected 1 month p.i; **D** - *O. felineus*-infected and SkQ1 treated 1 month p.i.; **E**- *O. felineus*-infected 3 months p.i; **F**- *O. felineus*-infected and SkQ1 treated 3 months p.i.
