# Supplementary material for: Plastoquinone-Derivative SkQ1 Improved the Biliary Intraepithelial Neoplasia during Liver Fluke Infection
Source: Curr Issues Mol Biol. 2024 Feb 17;46(2):1593–606. doi: 10.3390/cimb46020103 (PMC10887543; doi:10.3390/cimb46020103)
Supplement: Supplementary file 1 [file cimb-46-00103-s001.zip › Supplementary Table S1.pdf]

Supplementary Table S1. Primers and probes

**Primer**	**Sequences**
*Gapdh*_F	GAACATCATCCCTGCATCCACT
*Gapdh*_R	ATGCCTGCTTCACCACCTTCTT
*TNF-α*_F	TCTTCAGAGTCCATCTCCCTC
*TNF-α*_R	GGGAATTCTGGAAACCGTTTG
*Ck-7*_F	AGCTTCCCCGGATCTTTGAG
*Ck-7*_R	CGTCCTTCTTCAGCAACACAAAC
*Inos*_F	CATGACTTCCAGAACAGAGGG3
*Inos*_R	GCCATCTCCTGCATCTCTTC3
Inos_probe	R6G-CGGCACCCAAACACCAAGGTC-BHQ2
*Acta2*_F	GGATGCAGAAGGAGATCACAG
*Acta2*_R	TGGAAGGTAGACAGAGACGC
Acta2_probe	R6G-TTCATGGTGCTGGGTGCGAGA-BHQ2
*Alox5*_F	AGATCGTGGCTACTCTACCAG
*Alox5*_R	TCCTTCACTGGCTTCTCAATG
Alox5_probe	R6G-AATGCCCACACTGCGCCTAGA-BHQ2
*TGF-β*_F	CCAACTATTGCTTCAGCTCCAC
*TGF-β*_R	CAGGGCCAGGACCTTGCT
