# Supplementary material for: Plastoquinone-Derivative SkQ1 Improved the Biliary Intraepithelial Neoplasia during Liver Fluke Infection
Source: Curr Issues Mol Biol. 2024 Feb 17;46(2):1593–606. doi: 10.3390/cimb46020103 (PMC10887543; doi:10.3390/cimb46020103)
Supplement: Supplementary file 1 [file cimb-46-00103-s001.zip › Supplementary Table S2.pdf]

Supplementary Table S2. The results of the semiquantitative histological analysis

	Group	Biliary neoplasia mean ± SD	Epithelium hyperplasia mean ± SD	Periductal fibrosis mean ± SD	Inflammation mean ± SD	Parenchyma dystrophy mean ± SD	Bile duct proliferation mean ± SD
1 month	Uninfected	0	0	0	0.1	0.1 ± 0.02	0
	SkQ1	0	0	0	1.2 ±1.9	0.4 ± 0.06	0
	OF	0	8.7 ± 1.8***	46.7±7 ***	14.4±1.3 ***	19.3±2.7***	14.1±0.9 ***
	OF+ SkQ1	0	0.6±0.3** ###	22.4±13.8 ***	13.2±11*	9.7±3.5*** ##	10.2±7.8**

	Group	Biliary neoplasia mean ± SD	Epithelium hyperplasia mean ± SD	Periductal fibrosis mean ± SD	Inflammation mean ± SD	Parenchyma dystrophy mean ± SD	Bile duct proliferation mean ± SD
3 months	Uninfected	0	0	0	0,1	0.1 ± 0.02	0
	SkQ1	0	0	0	0,1	0.4 ± 0.06	0
	OF	3.3±0.8 ***	8.3±1.3 ***	46.1±3.1 ***	24.8±2.3 ***	30 ± 2.1***	15.9±2.9 ***
	OF+ SkQ1	2±0.3 ** ##	5±0.6 *** ##	42±2.7 ***	15.1±1.5 *** ##	14.3 ± 1.72 *** ##	5.4±0.7 *** ##

SkQ1- uninfected group treated with SkQ1; OF - group infected with *O. felineus*; OF+ SkQ1 - infected group treated with SkQ1;

For histopathological analysis, the tissue slides were stained with hematoxylin and eosin or Masson's Trichrome dye by the standard methods roscope. Histological features of liver were manually scored by two independent investigators and a senior pathologist confirmed the score. A scoring method of type Ratio (morphometry) of data measurements was applied [20]. This method is based on counting several fields of tissue (e.g. ten random 400x fields) for each animal, each field scored and a mean score assigned for the whole tissue of that animal. Each slice of a lobe was analyzed in all fields of view (20–30 fields). Each field of view was divided into 100 squares. Inflammatory cell infiltration, cholangiocyte hyperplasia, Biliary neoplasia, periductal fibrosis, and bile duct cell proliferation were assessed by means of a percentage of the area (the number of squares occupied).

P values were obtained by the Mann–Whitney U test. * Difference between groups (treatment/infected vs. uninfected), $ difference between groups (treatment vs. infected group), *# p< 0.05; **## p< 0.01 ***### p< 0.001; *-confirm to intact group; #-confirm to infected group.
