# Supplementary material for: Plastoquinone-Derivative SkQ1 Improved the Biliary Intraepithelial Neoplasia during Liver Fluke Infection
Source: Curr Issues Mol Biol. 2024 Feb 17;46(2):1593–606. doi: 10.3390/cimb46020103 (PMC10887543; doi:10.3390/cimb46020103)
Supplement: Supplementary file 1 [file cimb-46-00103-s001.zip › Supplementary Table S3.pdf]

Supplementary Table S3. Serum biochemistry

	**Reference**	**Uninfected,** mean ± SD	**SKQ1,** mean ± SD	**Infected,** mean ± SD	**Infected + SkQ1,** mean ± SD
	**1 month**				
**ALT**, u L-1	38.0 ± 26.1	35.9 ± 10.3	38.7±3.3	247.8 ± 67.1* p=0.02	204.3 ± 77
**AST**, u L-1	47.0 ± 38.3	51.5 ± 8.7	43.4±8.4	87.1 ± 30.4	53.7±12
**Total bilirubin**, µmol L-1	3.4 - 12.6	6.9 ± 4.2	11.6±2.5* P=0.03	24.7 ± 5.6 * P=0.02	16.4±3.4
**Total cholesterol**, mmol L-1	2.9 - 5.4	1.6 ± 0.2	2±0.4	2.9 ± 0.5 * P=0.02	3.9±2
**Triglycerides**, mmol L-1	0.8 - 2.6	1.0 ± 0.3	0.8±0.4	1.1 ± 0.1	1.8±0.2** P=0.009
**Urea**, mmol L-1	3.2 ± 0.7	4.7 ± 1.6	8.8±1.2* P= 0.02	5.1 ± 1.0	5.4±1.1
**Creatinine**, µmol L-1	90.1 ± 23.4	53.8 ± 45.6	89.7±22.2	112 ± 49.8	110.5±98.8
	**3 months**				
**ALT**, u L-1	38.0 ± 26.1	35.9 ± 10.3	45.7±16.5	356.7 ± 150.9*	212.2±77
**AST**, u L-1	47.0±38.3	51.5±8.7	53±22.3	95.0±37.6 * P=0.03	83.3±20.5
**Total bilirubin**, µmol L-1	3.4 - 12.6	3.4 ± 1.6	3.3±0.3	9.4 ± 6.1 ** P=0.01	5.9±1.6
**Total cholesterol**, mmol L-1	2.9 - 5.4	1.6 ± 0.2	1.7±0.1	2.9 ± 0.5 ** P=0.01	2.2±0.5
**Triglycerides**, mmol L-1	0.8 - 2.6	0.46 ± 0.42	1±0.5	0.67 ± 0.12	2±0.8$$* P=0.01
**Urea**, mmol L-1	3.2 ± 0.7	7.2 ± 1.0	7.4±0.4	5.7 ± 1.2	6.7±2.4
**Creatinine**, µmol L-1	90.1 ± 23.4	131 ± 81	67.3±44.6	83.8 ± 45.3	87.7±53

P values were obtained by the Mann–Whitney U test. * difference between groups (treatment vs. control), $ difference between groups (treatment vs. infected group), *$ p< 0.05; **SS p< 0.01 # parameter values taken from (Van Hoosier, G.L., McPherson, C.W., 1987. Laboratory hamster. Academic Press Inc., New York) Abbreviation: SD, standard deviation; ALT, Alanine transaminase; AST, Aspartate aminotransferase
